# Esophageal Self-Expandable Metal Stents Can Fracture in the Distal Third When Used for Post-Bariatric Surgery Complications: A Single Center Experience and Review of the Literature with Video

**DOI:** 10.3390/jcm13123419

**Published:** 2024-06-11

**Authors:** Yazan Abboud, Mohamad Othman El Helou, Joseph Meza, Jamil S. Samaan, Liliana Bancila, Navkiran Randhawa, Kenneth H. Park, Shahab Mehdizadeh, Srinivas Gaddam, Simon K. Lo

**Affiliations:** 1 Karsh Division of Gastroenterology and Hepatology, Cedars-Sinai Medical Center, Los Angeles, CA 90048, USA; mohamadosman.helou@lau.edu (M.O.E.H.); joseph.meza@ucla.edu (J.M.); jamil.samaan@cshs.org (J.S.S.); liliana.bancila@cshs.org (L.B.); kenneth.park@cshs.org (K.H.P.); shahab@cshs.org (S.M.); srinivas.gaddam@cshs.org (S.G.); 2 Department of Internal Medicine, Rutgers New Jersey Medical School, Newark, NJ 07103, USA; 3 Department of General Surgery, Yale University, New Haven, CT 06510, USA; 4 Division of Gastroenterology and Hepatology, Augusta University, Augusta, GA 30912, USA; randhnk@gmail.com

**Keywords:** self-expandable metal stent, stent complication, stent fracture, endoscopy, esophagus

## Abstract

**Background:** Esophageal self-expandable metal stents (SEMS) are an important endoscopic tool. These stents have now been adapted successfully to manage post-bariatric surgery complications such as anastomotic leaks and strictures. In centers of expertise, this has become the primary standard-of-care treatment given its minimally invasive nature, and that it results in early oral feeding, decreased hospitalization, and overall favorable outcomes. Self-expandable metal stents (SEMS) fractures are a rare complication of unknown etiology. We aimed to investigate possible causes of SEMS fractures and highlight a unique endoscopic approach utilized to manage a fractured and impaled SEMS. **Methods:** This is a retrospective study of consecutive patients who underwent esophageal SEMS placement between 2015–2021 at a tertiary referral center to identify fractured SEMS. Patient demographics, stent characteristics, and possible etiologies of fractured SEMS were identified. A comprehensive literature review was also conducted to evaluate all prior cases of fractured SEMS and to hypothesize fracture theories. **Results**: There were seven fractured esophageal SEMS, of which six were used to manage post-bariatric surgery complications. Five SEMS were deployed with their distal ends in the gastric antrum and proximal ends in the distal esophagus. All stents fractured within 9 weeks of deployment. Most stents (5/7) were at least 10 cm in length with fractures commonly occurring in the distal third of the stents (6/7). The wires of a fractured SEMS were embedded within the esophagogastric junction in one case, prompting the use of an overtube that was synchronously advanced while steadily extracting the stent. **Discussion:** We suggest the following four etiologies of SEMS fractures: anatomical, physiological, mechanical, and chemical. Stent curvature at the stomach incisura can lead to strain- and stress-related fatigue due to mechanical bending with exacerbation from respiratory movements. Physiologic factors (gastric body contractions) can result in repetitive squeezing of the stent, adding to metal fatigue. Intrinsic properties (long length and low axial force) may be contributing factors. Lastly, the stomach acidic environment may cause nitinol-induced chemical weakness. Despite the aforementioned theories, SEMS fracture etiology remains unclear. Until more data become available, it may be advisable to remove these stents within 6 weeks.

## 1. Introduction

Esophageal self-expandable metal stents (SEMS) are an important endoscopic tool [1]. One of the common uses of these stents is in the treatment of esophageal strictures [2]. These stents have now been adapted successfully to manage post-bariatric surgery complications such as anastomotic leaks and strictures [3]. In centers of expertise, this has become the primary standard-of-care treatment given its minimally invasive nature, and that it can result in early oral feeding, decreased hospitalization, and overall favorable outcomes [4].

SEMS are made of a nitinol or stainless-steel wire mesh and can be uncovered, partially covered, or fully covered. They are the stents of choice for esophageal and gastric sleeve leaks [5]. They are also used in the management of post-bariatric surgery esophageal and gastric strictures, with growing data showing their feasibility and safety. They can help in alleviating dysphagia whether from benign or malignant etiologies. Complications associated with these stents can be categorized into three groups depending on the time of occurrence, as follows: early complications (include intraprocedural issues such as aspiration and perforation), postprocedural complications (such as chest pain, bleeding, and airway compression), and delayed complications (that occur outside of the periprocedural period such as stent migration, obstruction, or food impaction, and rarely, fistula formation) [6].

Furthermore, malfunction and breakage of the device can lead to additional complications. Some of these complications include mucosal damage, ulceration, and bleeding. Other complications include impalement of the fractured parts of the stents in the mucosa, leading to pain, dysphagia, and even sometimes obstruction. Considered rare events, the etiology of stent fractures is not well known. The surprising observation of multiple cases of stent fractures has prompted us to examine this issue more carefully. Here, we report on seven cases of esophageal SEMS that fractured when placed into the stomach. We aim to further explore the possible causes of this rare complication. In addition, we describe a unique endoscopic approach to managing an impaled, fractured SEMS.

## 2. Materials and Methods

This is a retrospective study of consecutive patients who underwent esophageal SEMS placement between August 2015 and December 2021, with the goal of identifying fractured SEMS. Patient demographics, stent characteristics, and possible etiologies of fractured SEMS were identified. A comprehensive literature review was also conducted to review all data on prior fractured esophageal SEMS. Prior studies were also reviewed to investigate the differences in presentation, diagnosis, and management modalities of fractured stents. The study was approved by the institutional review board (IRB) committee.

## 3. Results

During the study period, there were 134 esophageal SEMS that were mainly inserted for gastric indications, of which 119 stents were used for post-bariatric surgery complications. There were seven cases of esophageal SEMS that fractured when they were deployed in the stomach for post-bariatric surgical complications (gastric stricture or leak) or to manage GERD-related strictures. These fractured stents were placed with the distal end in the stomach and the proximal end in the esophagus. Most of the fractures (6/7) occurred in the distal third of the stents. All stents fractured within nine weeks after deployment. Herein, we present the seven cases of fractured SEMS.

**Figure 1:**
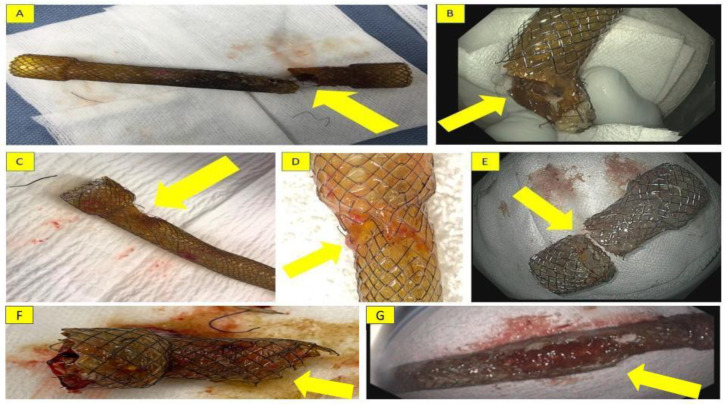
Fractured esophageal fully covered self-expandable metal Stents (SEMS): (**A**) case 1: complete fracture of an esophageal fully covered SEMS at the distal third of the stent; (**B**) case 2: partial fracture of an esophageal fully covered SEMS at the distal third of the stent; (**C**) case 3: partial fracture of an esophageal fully covered SEMS at the distal third of the stent; (**D**) case 4: partial fracture of an esophageal fully covered SEMS at the distal third of the stent; (**E**) case 5: complete fracture of an esophageal fully covered SEMS at the distal third of the stent; (**F**) case 6: partial fracture of an esophageal fully covered SEMS at the middle third of the stent; and (**G**) case 7: partial fracture of an esophageal fully covered SEMS extending from the middle third to the distal third of the stent. The arrows point to the fractures.

Case 1:

A 22-year-old morbidly obese female who underwent laparoscopic sleeve gastrectomy (LSG) presented three days following discharge with fever and abdominal pain. An abdominal CT demonstrated a gastric anastomotic leak. An 18 mm × 160 mm fully covered nitinol SEMS (Bonastent, Standard Sci-Tech Inc., Seoul, Republic of Korea) was placed, with the distal end situated in the antrum and the proximal end in the distal esophagus. Six weeks later, the stent was removed; however, a complete fracture was noted at its distal third when examined ex vivo (Figure 1A). The remaining distal portion was partially embedded in the gastric wall and was subsequently removed successfully.

Case 2:

A 44-year-old morbidly obese male presented to the ED ten years after laparoscopic gastric banding with vomiting and abdominal pain. He was found to have a slipped laparoscopic band, which was removed. Three weeks following discharge, the patient was readmitted due to fever and abdominal pain. An upper gastrointestinal series revealed a gastric anastomotic leak. The patient underwent EGD with placement of an 18 mm × 160 mm fully covered nitinol SEMS (Bonastent, Standard Sci-Tech Inc., Seoul, Republic of Korea) with the distal end in the antrum and the proximal end in the distal esophagus. Six weeks later, the stent was removed but a partial fracture was noted in its distal end (Figure 1B).

Case 3:

A 65-year-old morbidly obese female who underwent removal of a laparoscopic gastric band and LSG was readmitted one week following discharge with purulent discharge through the abdominal wall. An EGD revealed a proximal anastomotic gastric leak; an 18 mm × 160 mm fully covered nitinol SEMS (Bonastent, Standard Sci-Tech Inc., Seoul, Republic of Korea) was placed in the antrum with the proximal end in the distal esophagus. Six weeks later, she presented with bilious vomiting. An EGD showed the stent had migrated distally to the stomach and a partial fracture of the distal third of the stent had occurred (Figure 1C). The free ends of some wires at the fracture were embedded into the esophagogastric junction during stent withdrawal, leading to stent fixation. Advancing the scope between the stent and esophageal wall to free the impaled part of the stent was unsuccessful. The scope was then advanced through the stent to push it distally into the stomach to free it up. An overtube was inserted into the stomach to prevent the wires from coming into contact with the gastric and esophageal mucosa. The proximal string of the stent was grasped, and the stent was pursed. Steady traction on the forceps combined with advancing the overtube resulted in a smooth delivery of the stent through the overtube without any complications (Video S1).

Case 4:

A 31-year-old morbidly obese female who underwent LSG presented to the ED two months post-operatively with abdominal pain and intractable vomiting. An upper gastrointestinal series revealed a mid-gastric stricture with possible twisting and angulation. An 18 mm × 160 mm fully covered nitinol SEMS (Bonastent, Standard Sci-Tech Inc., Seoul, Republic of Korea) was placed across the stricture and secured proximally by a StentFix device against the distal esophagus. Four weeks later, the patient presented with abdominal pain, hematemesis, and melena. She was found to have granulation and ulceration in the distal antrum, likely due to stent irritation, which was noted to have been partially fractured at its distal third (Figure 1D).

Case 5:

A 42-year-old morbidly obese female presented 3 months after an LSG with persistent dysphagia, early satiety, and postprandial regurgitation. An upper gastrointestinal series revealed a 2 cm mid-gastric stricture, treated by an 18 mm × 60 mm fully covered nitinol SEMS (Bonastent, Standard Sci-Tech Inc., Seoul, Republic of Korea). Due to persistent vomiting and abdominal discomfort, the patient requested the stent to be removed, which was done at 7 weeks post-placement. The distal 2 cm portion of the stent was found freely floating in the antrum (Figure 1E).

Case 6:

An 81-year-old male with a history of GERD-related stricture at the gastroesophageal junction presented with recurrent dysphagia despite previous dilation. Placement of a 60 mm × 18 mm fully covered nitinol SEMS (Hanarostent, Olympus America Inc., Webster, TX, USA) was performed, with the distal end in the cardia above the diaphragmatic pinch and the proximal end in the distal esophagus; the stent was sutured in place. Nine weeks later, the patient presented with dysphagia. An EGD revealed impalement of stent in the esophageal mucosa and a partial fracture beyond its upper flange at the middle third (Figure 1F). The sutures were cut, and attempts were made to remove the stent in a standardized fashion but were unsuccessful. Therefore, the proximal part of the stent was inverted inwardly, the distal part was subsequently grabbed with rat-tooth forceps, and the stent was pushed distally into the stomach. A gastric overtube was then inserted into the stomach, and the stent was removed through it.

**Figure 2:**
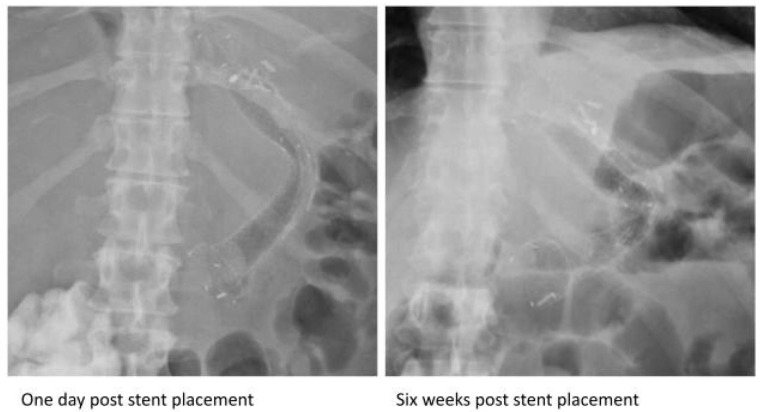
Subsequent radiographs after the placement of the esophageal self-expandable metal stent in the seventh case demonstrating acute angulation at the same location of the partial fracture.

Case 7:

A 45-year-old morbidly obese male presented seven months after an LSG with fevers and was diagnosed with a gastric anastomotic leak and mid-body gastric stricture. A small pigtail plastic stent was placed into the fistula and was clipped to prevent migration. Thereafter, a 150 mm × 20 mm fully covered nitinol SEMS (Hanarostent, Olympus America Inc., Webster, TX, USA) was placed, with the distal end in the antrum and the proximal end in the distal esophagus. Six weeks later, an abdominal radiograph revealed kinking at the midpoint of the stent (Figure 2). An EGD revealed a fracture extending from the middle third of the stent to its distal end, with wires pointed out (Figure 1G).

## 4. Discussion

In addition to the cases presented, there have been 23 previously reported cases of complete fractures [7,8,9,10,11,12,13,14,15,16,17,18,19,20,21,22,23,24] and 37 of partial fractures [9,25,26,27,28,29,30,31,32,33]. A summary of patient demographics and stent characteristics is shown in Table 1. The exact cause of this fracture remains unknown. Several potential reasons have been cited, including stent-related causes such as spontaneous fracture [9,10,11,24,28,31], defective material or design [10,11,18,27,30], corrosion effect [12,19,23,25], and the embedment of the uncovered part of a partially covered stent [21], and iatrogenic-related causes such as tumor ablation through a metal stent using Nd: YAG laser application, which can cause thermal overstraining [28], stent removal attempts [22], and post-deployment balloon dilatation [27]. We propose additional plausible etiologies that can be categorized into anatomical, physiological, mechanical, and chemical etiologies.

### 4.1. Anatomical and Physiological Etiologies of SEMS Fracture

**Figure 3:**
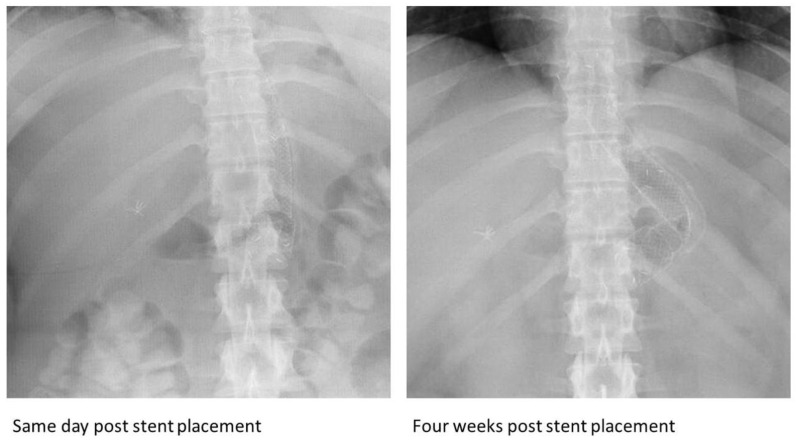
Subsequent radiographs after the placement of the esophageal fully covered self-expandable metal stent in the first case, demonstrating acute angulation and a possible twist at the same location of the complete fracture.

**Figure 4:**
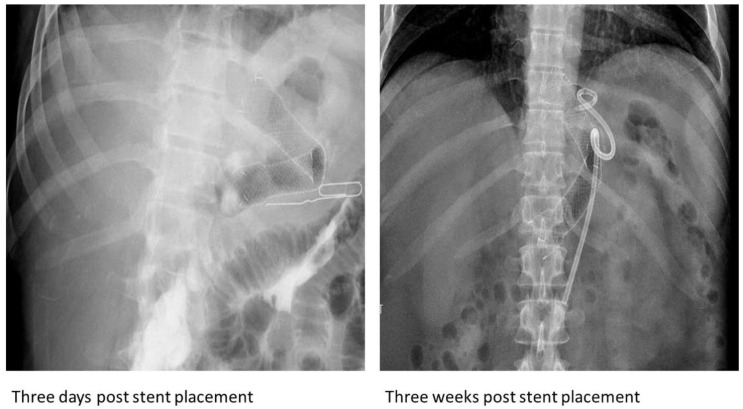
Subsequent radiographs after the placement of the esophageal self-expandable metal stent in the second case, demonstrating acute angulation in the distal third at a nearby location of the partial fracture.

**Figure 5:**
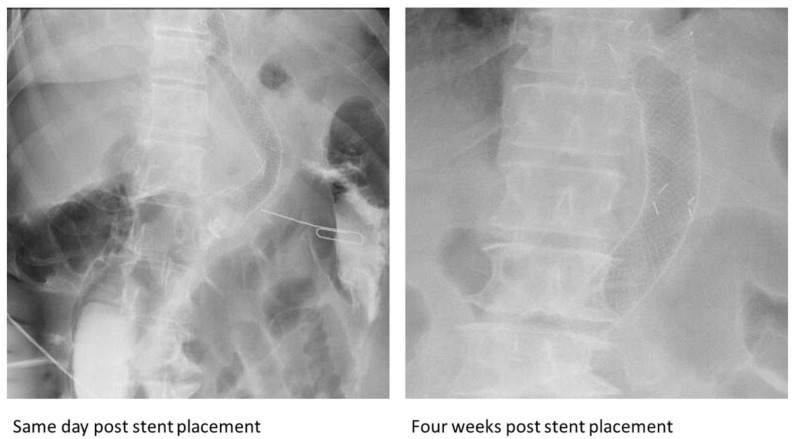
Subsequent radiographs after the placement of the esophageal self-expandable metal stent in the third case, demonstrating acute angulation in the distal third at a nearby location of the partial fracture.

The metal used in esophageal SEMS is often nitinol, as were all the stents in our case series, due to its ability to maintain its shape (metal memory) and its super elasticity, which allows for the restoration of the stents’ initial shape despite physiological and anatomical factors [34]. However, stents can sometimes deform when faced with persistent strain- and stress-related fatigue. This is often precipitated in situations where the stents remain curved in vivo, as in the case of being curved around the angle of the stomach at the incisura, and due to consistent cyclic loading on the stent [35] such as esophageal peristaltic movements and gastric body contractions. When stents are at the gastroesophageal junction, repetitive respiratory movements can result in recurrent bending at the angle of the stomach. The distal third of the stent is located at the angle of the stomach and can undergo recurrent stress in the metal in this area. This disproportionate stress and strain at the distal third, compared to other parts, can potentially lead to stent fractures such as those observed in cases 1 (Figure 3), 2 (Figure 4), 3 (Figure 5), and 7 (Figure 2).

Furthermore, physiologic gastric body contractions can move the stents distally, further aggravating their acute angle. This was especially evident in our 4th and 5th cases, where the patients had gastric body strictures and severe angulation that potentially increased the stress on the stents with each contraction. Moreover, when the distal third of the stent is deployed in the cardia of the stomach, repetitive respiratory movements can increase the angulation of the stent at the gastroesophageal junction, with the stent hitting the diaphragmatic pinch with each respiration. This may potentially increase the risk of fracture, as seen in our 6th case.

### 4.2. Mechanical Etiologies of SEMS Fracture

Other intrinsic stent characteristics, such as length, may also be related to fracture risk. The moment of force or turning effect is a measure of the force required for an object to turn about a pivot point [36]; this can explain why longer stents need less force to generate an angulation when compared to shorter stents, thereby leading to an increased risk of fractures. This was not only demonstrated in our cases but also in previous reports where, out of 24 stents with known lengths, 22 were at least 10 cm [7,8,9,10,11,12,15,18,20,25,26,28,31,32].

In addition to stent length, axial force may also play a role. Axial force is exerted by the stent to restore its natural straight shape after being deployed in a bent position [37]. This was extensively studied by Hirdes et al., who identified the axial forces of a variety of esophageal stents [38]. SEMS with the lowest axial forces were found to have fractured in about 31 cases (out of 65 reported cases) [29]. In contrast, SEMS with the highest axial forces were not found to be associated with any of the reported fracture cases. To that effect, Kadokura et al. recommended the use of shorter stents with higher axial forces to avoid fractures in duodenal SEMS [39]. However, Hirdes et al. express concern that stents with higher axial force can cause luminal wall injury [38]. Therefore, it is suggested that lower axial force SEMS are more appropriate in areas of anatomical bending, such as the hilar biliary strictures or malignant duodenal stricture, to avoid wall damage [40]. Clinicians must balance the risk of wall injury with stent fracture when choosing SEMS.

Our cases demonstrated a shorter duration between stent placement and fracture diagnosis (mean of 6.7 weeks, range: 4 to 9 weeks) compared to the literature, after excluding iatrogenic causes (mean of 37 weeks, range: 1 day to 3 years). While the reason for the discrepancy is unclear, it may be explained by the past surgical history of our patients. In cases of anastomotic leaks, strictures may form after exposure of the gastric lumen to acid, which can increase pressure on the stent [41]. Additionally, healing of the leak may exacerbate pressure on the stent by the presence of scar tissue. On a histopathological level, myofibroblasts increase matrix production and lead to granulation tissue formation, scarring, and tissue contraction [42]. Furthermore, manufacturer defects may have also played a role in precipitating fractures, although it is difficult to be certain. If stent manufacturing defects are present, they may have been compounded by the physiologic movements and chemical weakness described above, ultimately resulting in fracture of the distal third of the stents in most of our cases. This may be supported by the findings from a study of 71 patients who underwent stenting due to gastric outlet obstruction, where a significantly higher fracture rate in the Bonastent group compared to the Wallflex group (13.3% vs. 0%, *p* = 0.03) [43] was observed, although the locations of the fractures were not reported. Further studies are needed to understand differences in intrinsic stent characteristics and the risk of fracture. Lastly, operator-induced factors, such as deployment technique and post-deployment manipulation, may be factors to be considered.

### 4.3. Chemical Etiologies of SEMS Fracture

Similar to previously reported cases, this case series reports the use of SEMS made of nitinol. Despite many favorable features, the manufacturing materials, fabricating method, and overall design may affect clinical performance and longevity [44,45]. Experimental studies have been conducted to investigate nitinol-induced chemical weakness. The reaction between nitinol and liquid surfaces results in hydrogen generation (H^+^), uptake, and diffusion through the nitinol matrix, leading to the formation of a crack and subsequent fracture of SEMS [46]. In addition, stent location can be a contributing factor to expedition of the chemical weakness. While our stents were deployed with their distal end in the antrum or cardia, most of the stents reported in the literature (18/21) were deployed at the distal esophageal third, potentially with their distal end in the stomach. We hypothesize that the stomach’s acidic environment, as previously explained, may have led to nitinol-induced chemical weakness affecting the distal parts of the stents. To that effect, six out of our seven cases fractured at the distal third. Interestingly, and compatible with our cases, a trend was observed in the literature regarding fracture location where 17/19 stents fractured at the distal third as well.

### 4.4. Clinical Presentation, Diagnosis, and Management of Fractured SEMS

A wide range of clinical presentations and management modalities of SEMS fractures are reported in the literature (Table 2). While most of the completely fractured cases presented as dysphagia, most of the partially fractured cases were asymptomatic. As for diagnosis, the true incidence of stent fractures is likely underreported, given that patients can be asymptomatic, especially in the case of a partial fracture. This likely results in the diagnosis of a fractured SEMS at the time of endoscopy. This can also lead to variation in the time to diagnose the fractures. Furthermore, when SEMS are placed for palliative treatment of malignant dysphagia, a patient’s death may occur prior to the development of symptoms or stent removal or exchange. Diagnostic methods in the literature and in our cases can also be seen in Table 2. Most of the fractured stents were diagnosed during, or after, endoscopic removal. With regard to the management of the fractured stent, most of the stents were managed endoscopically (Table 2). We report an endoscopic approach to retrieving an impaled stent using a gastric overtube (Supplementary Video S1).

### 4.5. Future Directions and Limitations

We recommend that endoscopists carefully evaluate the stent prior to removal for any fractures or embedded wires that may result in removal difficulty, mucosal damage, or worse, a perforation. Additionally, subsequent radiographs of esophageal SEMS post-deployment may help to detect any change in the curving pattern that can predispose fractures, with an intent to anticipate this complication and minimize the risk of mucosal damage at the time of removal.

Our study was limited by a small sample size. The indications for stent placement in our cases differ from the literature. However, we summarized all indications in Table 1. Moreover, hypothesized etiologies of fractures were theoretical and will need future in vivo and in vitro investigations to better characterize etiologies of SEMS fractures with the goal of designing stents resistant to physiological, anatomical, and environmental stress.

## 5. Conclusions

In conclusion, esophageal SEMS fracture is a rare and poorly understood complication. When esophageal SEMS are indicated to manage post-bariatric surgery complications, fracture of the distal third can occur within a relatively short period in longer stents with low axial forces. Despite the theories and the observed pattern in our cases that suggests potential causes of SEMS fracture in the distal third, fracture etiology remains unclear. SEMS manufacturers should balance stent length and its axial force when developing future stents that are meant to be deployed in curved anatomical locations. The novel endoscopic approach (Video S1) used to manage the fractured impaled stent by advancing an overtube while simultaneously extracting the stent can aid in avoiding perforations or stent-related mucosal injuries. Further investigations of esophageal SEMS fracture etiologies are needed, as they may aid endoscopists to avoid, or at least anticipate, this poorly understood complication.

## Figures and Tables

**Table 1:** Patient demographics and characteristics of fractured esophageal self-expandable metal stents (SEMS).

Fracture Type	Complete	Partial
Number of cases ^	25	42
Age [mean (±SD)]	60.8 (±17.2)	57.6 (±15.9)
Gender [N (%)]		
Male	9 (56.2%)	6 (60%)
Female	7 (43.7%)	4 (40%)
Duration of placement * [mean (±SD)] (weeks)	30 (±35.7)	27.35 (±35.8)
Indication [N (%)]		
Malignant dysphagia	13 (65%)	36 (83.7%)
Benign esophageal disease	5 (25%)	1 (2.3%)
Gastric anastomotic leak	1 (5%)	3 (7.0%)
Gastric anastomotic stricture	1 (5%)	2 (4.7%)
GERD-related stricture	0 (0%)	1 (2.3%)
Type of stent [N (%)]		
Fully covered	9 (42.8%)	9 (81.8%)
Partially covered	2 (9.5%)	1 (9.1%)
Uncovered	10 (47.6%)	1 (9.1%)
Stent location [N (%)]		
Proximal esophageal third	1 (6.2%)	0 (0%)
Middle esophageal third	1 (6.2%)	1 (9.1%)
Distal esophageal third	12 (81.2%)	6 (54.5%)
Distal esophageal third till the antrum	2 (6.2%)	3 (27.3%)
Distal esophageal third till the cardia	0 (0%)	1 (9.1%)
Approximate fracture location [N (%)]		
Proximal third	4 (18.1%)	1 (25%)
Middle third	3 (16.6%)	2 (50%)
Distal third	15 (68.0%)	1 (25%)

^ This table has data on all fractured stents from the current manuscript (2 complete fractures and 5 partial fractures) and from prior cases reported in the literature (23 complete fractures and 37 partial fractures) that sum up to a total of 67 stent fractures (25 complete fractures and 42 partial fractures). * One partial fracture case that occurred immediately post-deployment of the stent was not included. Data on patient’s demographics and stents’ characteristics included in the table are limited to only cases with known variables (i.e., there were only 26 cases out of the 67 cases with known gender). Percentages represent the number of variables divided by the total number of completely fractured or partially fractured stents with known data points.

**Table 2:** Presentation, diagnosis, and management of fractured self-expandable metal stents (SEMS).

**Presentation**			
**Complete SEMS Fracture**		**Partial SEMS Fracture**	
**Prior Literature**	**Our Cases**	**Prior Literature**	**Our Cases**
Dysphagia [7,10,11,12,18,20] Coffee ground emesis [10] Regurgation [11] Emesis of fractured Stent particles [21] Abdominal pain [24] Asymptomatic [8,19]	First case was asymptomatic while the fifth patient presented with nausea, vomiting and abdominal discomfort.	6 cases presented with dysphagia [25,28,29,31,32] 28 were asymptomatic [9,26,29,30]	Second and seventh cases were asymptomatic The remaining presented as abdominal cramping, melena, dysphagia, chest pain, or nausea and vomiting
Diagnosis			
Prior Literature		Our Cases	
Some cases were diagnosed by imaging [8,9,11,12,13,16,20,24,32] Most were diagnosed during or after endoscopic removal		All the SEMS fractured in vivo and were noted during or after endoscopic removal.	
Management			
Complete SEMS fracture		Partial SEMS Fracture	
Prior Literature			
3 patients reported in the literature developed small intestinal obstruction from migration of a fractured part of the stent and required surgical management [12,13,16] 1 developed a gastrocolic fistula also due to the migrated fractured part requiring surgical management [14] 2 passed the stent rectally [8,15] 10 stents were removed endoscopically [7,8,9,10,17,18,19,20,21,22] 2 were left in place [11,12]		2 were asymptomatic and were removed endoscopically after migration of the stents [30] 1 was also asymptomatic and was removed endoscopically after being diagnosed using a contrast enhanced CT scan [9] 2 in which dysphagia occurred were removed endoscopically [31,32] The remaining 23 partially fractured SEMS was not reported, and they were likely left in place given the majority were asymptomatic and working as intended [25,26,27,28,29]	
Our Cases			
All our cases underwent endoscopic removal. Our third and sixth cases were managed using a gastric overtube to ensure mucosal protection and prevent fluid leaks. Overtube-assisted retrieval of foreign bodies including esophageal stents has been reported prior [47,48]. However, the endoscopic technique used to manage the impaled SEMS in our third case was unique, as the overtube was synchronously advanced with stent extraction, resulting in a safe and smooth removal as demonstrated in Video S1. Moreover, the proximal part of the stent in the sixth case was inverted inwardly, allowing for safe downward pushing of the fractured stent into the stomach. These techniques can aid future endoscopists in safely removing a fractured stent from the stomach			

## Data Availability

The original contributions presented in the study are included in the article/supplementary material, further inquiries can be directed to the corresponding author.

## Supplementary Materials

The following supporting information can be downloaded at: https://www.mdpi.com/article/10.3390/jcm13123419/s1, Supplementary Video S1: Overtube Assisted Removal of Fractured Esophageal Stent.
